# A Treatise on Varicose Capillaries, as Constituting the Structure of Carcinoma of the Hepatic Ducts, and Developing the Law and Treatment of Morbid Growths. With an Account of a New Form of the Pus Globule

**Published:** 1839

**Authors:** 


A TREATISE ON VARICOSE CAPILLARIES, AS CONSTITUTING THE STRUCTURE OF CARCINOMA OF THE HEPATIC DUCTS, AND DEVELOPING THE LAW AND TREATMENT OF MORBID GROWTHS. WITH AN ACCOUNT OF A NEW FORM OF THE PUS GLOBULE. By *Thomas Gordon Hake*, M.D. late Physician to the Brighton Dispensary, Orphan Asylum, &c. Quarto, pp. 20. London, Taylor and Walton, 1839.

SIR W. HERSCHEL observes in his Discourse on the Study of Natural Philosophy, that :—

"In a few instances only have we arrived at those general axiomatic laws which admit of direct deductive inference, and place the solutions of physical phenomena before us as so many problems, whose principles of solution we fully possess, and which require nothing but acuteness of reasoning to pursue even into their farthest recesses. In fewer still have we reached that command of abstract reasoning itself which is necessary for the accomplishment of so arduous a task. Science, therefore, in relation to our faculties, still remains boundless and unexplored, and after the lapse of a century and a half from the æra of Newton's discoveries, during which every department of it has been cultivated with a zeal and energy which have assuredly met their full return, we remain in the situation in which he figured himself,—standing on the shore of a wide ocean, from whose beach we may have culled some of those innumerable beautiful productions it casts up with lavish prodigality, but whose acquisition can be regarded as no diminution of the treasures that remain."

Do our readers deem us sanguine in applying these observations to medicine? We scarcely think ourselves so. Our attention has been chiefly turned, our acuteness principally directed, to the consequences and the symbols of disease, of the most obvious character—to symptoms and to structural changes which the unassisted sense of vision could perceive. How gross those must be, how general, how removed from the simpler and more elementary features of morbid action, need not, we conceive, be pointed out. The microscope is to be applied to the solids and the fluids in disease, and to distinguish, as no doubt it will, the primary deviations from normal mechanical constitution, which disease must of necessity exhibit, and in which it may often, perchance, consist. And chemistry is to come with her wonderful analysis, outrunning the microscope far more than the microscope outruns the eye. When we contemplate the field of research before us, we cannot think ourselves sanguine in anticipating a fertile crop of discovery in the future.

Dr. Hake informs us, in an advertisement, that:—

"I first saw varicose capillaries in tubercle of the spleen, given me, about ten months ago, at Paris, by M. Andral; I afterwards saw them in softening of the spleen, in which case they gave rise to hypertrophy of the entire organ, including the Malpighian bodies. In a preparation of intestine, which I was invited by Mr. Kiernan to inspect, I recognised the same state of the vessels, and communicated to Mr. Kiernan my opinion of its nature; but I had no suspicion of the full importance of this form of disease until recently, when I found that it was identical with carcinoma. The history of this discovery will be given in another place."

It is unnecessary to inform our readers that this statement has called up an indignant remonstrance and an angry denunciation of piracy from Mr. Kiernan. It would be foreign to our purpose to enter on the question of priority in this investigation, and in the discovery which hangs by it. Not that we think that question, personal as it is, an unimportant one. Scientific inventors should not be pillaged, nor should the laurel be basely filched or rudely torn from their brows. The encouragements to pursue abstract science are too few already. Reputation is almost the only recompense of those who woo it with success. Deprive them of that, and you strip them of all. The philosophic world is interested in maintaining a vigilant police, and *its* property must be guarded as jealously and as vigorously, as the goods and chattels of the citizen. We hold that the literary robber, and the scientific swindler, are as dangerous and as despicable as the ruffians or vagabonds of every-day life, as much to be discouraged by the honest expression of public opinion, and as openly to be denounced by the " hue and cry" of criticism. If, then, we do not interfere, at present, in this controversy, it is not because we esteem it insignificant, but because the data for judgment are imperfect. We shall content ourselves with simply detailing the observations of Dr. Hake, postponing to a future period all collateral considerations.

We do not anticipate that we shall be able to analyse the observations of our author. They are in too condensed a form. Where we can compress we will.

"There is a disease of the liver in the rabbit which consists of carcinomatous enlargement of the ducts, but manifests itself under the apparent form of small abscesses. This appearance is owing to dilatations of the ducts, which occur at regular intervals. These dilatations may be either few in number, or so numerous as to occupy the greater portion of the substance of the liver, and to be seen, through the peritoneum, to cover numerous points of its surface.

I had been recently engaged in investigating the characters of pus, as developed under the various aspects of disease, and the first step of my inquiry into this affection of the ducts was to examine, by the aid of the microscope, the purulent fluid which they contained, although with no other expectation than of finding some known variety of the pus globule. This fluid, however, proved to be of a nature different to that of common matter; it consisted of ovate corpuscules, several times larger than the globules of ordinary pus. These corpuscules contained within their exterior oval capsule a central nucleus of a circular form, corresponding generally in size to the greatest transverse diameter of the exterior capsule, though varying from that to the entire length of the oval envelope. Within the central nucleus numerous molecules were imbedded, some of greater and others of less size than those which may be exposed in the common pus globule by means of the solvent action of strong acetic acid.

A formal examination of the general characters of the disease and its relations led me to observe that the gall-bladder, cystic duct, and ductus communis choledochus, as well as the hepatic ducts, were distended with the fluid. From the common duct it passed freely into the duodenum, with the contents of which as well as with those of the stomach and general intestines, it was mixed in considerable quantity. In two instances there was an obstruction to the passage of the fluid from the gall-bladder to the cystic duct. In one of these the gall-bladder was distended with a purulent, in the other with a greenish transparent fluid; but the ovate corpuscules were found within it in both cases.

The next step of the investigation was to ascertain the distribution of the vessels, and the means by which this matter was deposited in the ducts; the external surface of the enlarged portions of which appeared highly vascular, and of a deep red colour.

Having obtained another rabbit, I tied the aorta and portal vein so as to cut off all supply of blood to the liver. I then opened the hepatic vein. The liver, which was occupied by the disease to the fullest extent, was carefully injected on the following day, and with perfect success. The highly vascular surface of the ducts received the injection from both the hepatic artery and portal vein, but principally from the latter. The white fluid within the ducts was in some parts tinged with green, the colour employed to inject the portal vein; in others with the red injection from the artery. On opening different ducts and examining their inner surface, I found it to be the seat of a thick, irregular, morbid growth, which was deeply impregnated with the red and green injections, but in which no vessels were detectable by the naked eye, except the more considerable branches of the hepatic artery.

Having subjected both the outer or vaginal, as well as the inner surface of these highly-injected ducts, to microscopic observation, I found the former covered with an exceedingly minute plexus, derived from the portal vein, with branches of the artery crossing it. This plexus in many parts possessed the characters of healthy vessels; a fact of the highest interest in physiology, since it shows the exact manner in which the portal circulation is carried to the hepatic ducts; and being continued to the inner surface of the ducts, as will be presently shown, it explains the secretion of bile on the same principle as that of other fluids, viz. by the distribution of vessels over a free surface.

An examination of the inner surface of the ducts proved that the new growth consisted of a plexus of capillaries derived from the portal vein, interspersed with others from the hepatic artery. This plexus, however, instead of presenting a healthy form, was seen to consist of varicose vessels, in some parts dilated to an extraordinary degree and knotted, and in others contracted. Indeed, the entire structure consisted of a plexus of varicose capillary vessels." 7.

### *Varicose Capillaries the efficient Cause of Carcinoma.*

Dr. Hake pays a compliment to the accuracy of Dr. Carswell:—

"Dr. Carswell, in his elegant Illustrations of the Elementary Forms of Disease, has supplied a drawing of the affection of which this paper treats, and has the entire merit of having ascertained that it existed in the ducts, the extremities of which, he says, 'are dilated into the form of pyriform sacs of various sizes. In these sacs, as well as in the biliary ducts, the tubercular matter was of the colour and consistence of cream, and was made to flow out at the cut extremity of the ductus communis choledochus, when pressure was applied either to the hepatic ducts or to their bulbous extremities. The pyriform sacs presented externally a smooth uniform surface; except in two or three instances, in which they were slightly lobulated. Internally, some of them had a cellular aspect, but the most of them were uniformly hollow. The fundus of several was connected with the substance of the liver by what appeared to be small blood-vessels.'" 7.

Dr. Hake has confirmed these observations by examination of the disease in its early stages.

At first, he says, simple dilatation of the duct, without thickening, takes place; the bile found within the duct at this period of the disease contains globules like those of the blood, but of three or four times their diameter, and occasionally with a molecule formed on their disc. In a more advanced stage the duct is thickened and pyriform at its egress from the glandular grains of the liver, and contains pus. This thickening of the duct represents the crude state of carcinoma; but as the disease advances, and the whole of the ducts become knotted, and indeed varicose, the structure softens, and presents all the characters of medullary sarcoma. The cellular aspect which the disease presents is caused by shoots of vessels in the form of flocculi; and its ultimate softening by the tenuity of the vessels, which increases with their varicose growth.

It happens frequently that the outer surface of the enlarged ducts is coloured, from the presence of congested vessels. By injection, this appearance is proved to be a plexus of capillaries from the portal vein. These capillaries not being varicose to the same extent as those within the duct, but often of a regular form and diameter, may be supposed to represent the distribution of the portal vein to the hepatic ducts in their healthy state. Branches of the artery also cross these ducts; but their plexiform distribution is reserved for the inner surface, where they blend, without anastomosing, with a similar structure of portal capillaries.

The vessels which connect the enlarged ducts and the glandular grains of the liver are the minute ducts, which are distributed round those bodies. The walls of these capillary ducts, which do not participate in the disease, as well as those of the larger branches, receive injection from the portal vein.

The carcinomatous structure of the ducts is entirely vascular, and consists of a plexus of varicose capillary vessels, which extends in every direction, and not over the surface alone. In fact, the diseased vessels commencing from the external plexus anastomose, and proceed to the inner surface of the ducts, while, in the interstices which they leave between them, the pus is collected. The varicose state of the capillaries is almost coetaneous with their formation, the process of their growth being subject to pathogenic laws, if not their first formation. The new vessels do not shoot into an albuminous or any other deposit, as some have supposed; but proceed in the form of flocculi from a vascular base, become varicose, and again give out branches; and their increase alone constitutes the growth of carcinoma, and their different degrees of varix its progressive softening.

Such is the manner in which this morbid growth is developed within the ducts. Its structure is entirely vascular. If a transverse section of the duct be made, the plexus of varicose capillaries which forms its substance is seen to resemble a honeycomb; and the vessels are so minute, that only the more dilated ones are seen when magnified from twenty to thirty times linear.

The capillaries of the hepatic artery, though fewer in number than the venous, are equally the seat of varicose enlargement. And Dr. Hake summarily states that the efficient cause, then, of carcinoma, as existing in the hepatic ducts, is to be found in universal varix of the veins and aneurysm of the arteries, as affecting capillaries, and in the simple increase of vessels so affected.

### *Comparison of the Pus Globule, the Blood Globule, and the Matter contained in Diseased Hepatic Ducts.*

"The purulent matter," we are still quoting Dr. Hake, "found within the diseased hepatic ducts of the rabbit, when examined by the assistance of the microscope, is seen to consist of two principal parts, a serous fluid and ovate corpuscules. The latter bodies bear the same relation to their containing fluid, as the blood or pus globules to their serum. They are semi-transparent: in form they resemble ova, but cannot be considered as such for various reasons. In ova there is a cohesion among the various parts, a continuity of organization: a state altogether contrary to this is seen in the ovate corpuscules, the great feature of which is their tendency to degrade from the normal form, and be resolved into their constituent parts, the relations between which are entirely physical. The substance of the ovate corpuscule is homogeneous, each of its parts being complete in itself, having a uniform structure, and united to the rest only through contiguity of substance and not by continuous organization. The membranous capsules are constantly seen disengaged from their nuclei; the latter are found without their ovate capsules, while perfect in every other respect. Again, these circular nuclei, which differ in size, whether contained within or disengaged from their capsule, are sometimes partially or entirely freed of their molecules, while the latter, either single or in groups, remain in the serum unaltered. Fragments of the ovate capsules also abound in the same medium, under various though oftentimes regular forms. These changes correspond, in a great measure, with those experienced by the ordinary pus globule, which, if observed in various diseases, will often be found disintegrated, whether in the blood or elsewhere, their molecules being discovered without their matrix, as in the case of the ovate corpuscules, and floating among the other constituents of pus; and the matrix existing entire, split into portions which are still adherent, or resolved into amorphous parts. In one instance, a quantity of chalky matter, deposited in the calyx of the kidney, proved to be composed entirely of pus molecules. In this case pus globules existed plentifully in the blood. Again, although the molecules of the ovate corpuscules, which vary in size, are occasionally much smaller, they are as frequently of nearly the same diameter as the common pus molecule; and in each circular nucleus there appear one or two molecules of a much larger size. Acetic acid does not appear to act on the ovate corpuscules, except by the aid of slight pressure, which is most conveniently applied between two pieces of glass; and by the same means the pus globule is entirely dissolved. Such is the principal evidence which I have to offer of the identity of pus and the fluid contained in diseased hepatic ducts; not to omit the impossibility of distinguishing one from the other by the naked eye, a proof sufficient in itself where the microscope is not employed.

Although I have examined pus extensively in various diseases, and both in and out of the blood, my observations do not afford sufficient data on which to decide the method of its formation. It may be well, however to state so much of what I have ascertained as may tend to assist the present discussion. It is not known whether pus is a degradation of the blood globule or a new formation. However, in purulent matter, formed from an accident, it happened to me to find that the globules were exactly the size of those of the blood. There were some pure blood globules, and others resembling them both in magnitude and in the evenness of their circumference, but which had one, two or more of those characteristic marks of pus which point out the situation in which the molecules are either formed, or are in the progress of formation. There were others, of the same size, in which those marks were most distinct, and the edges rough: but in pus produced from changes operating on the general system, or by chronic disease of a part, the globule is from one-third to double the size of the blood globule; it is more flattened than the latter, and has a broken border. This kind of globule may be detected in the blood in all cases in which the system is affected by a local deposit of pus. In the last stage of phthisis the blood was found by Dr. Carswell and myself to contain no other globules than those of pus.

These facts would favour the belief that pus is formed from the blood; and it is a view which, in the present state of our knowledge, I am most disposed to adopt: but, if the ovate corpuscules be purulent, as there is good reason for admitting, there is this difficulty, that they are greater in diameter than many of the vessels composing the plexus before described. The knotted portions of these vessels, however, as well as those which appear to have arrived at their highest degree of dilatation, are considerably larger than these corpuscules; and, since the latter occupy the interstices of their plexus as well as the channel of the ducts, it is more than probable that they are formed in these reservoirs from the venous blood which principally fills them." 11.

That pus is formed from the blood is, we suppose, certain. But that it is formed without submitting to some intercurrent transformation by the capillaries does not seem, to our understanding, favoured by the fact that the pus globule is found in the blood in all cases of local deposit of pus. This goes to establish resorption, rather than primary formation of the purulent fluid.

*Size of the Corpuscules and Varicose Capillaries.*

The greatest diameter to which the varicose capillary veins arrive is the hundredth part of an inch; but they often vary from that to the four-hundredth of an inch in the same branch: but the minutest branches, those which appear of new growth, are as small as the five-thousandth of an inch. By a comparison of the size of the ovate corpuscules, which are about the eight hundred and fiftieth part of an inch in their shortest, and the four hundred and fiftieth of an inch in their longest diameter, with the size of the larger vessels, it will be seen that the latter are sufficiently capacious to contain them.

The following speculations appear to us to stand upon stilts. We do not see, in the facts related, any premises which naturally lead to such conclusions. It is unnecessary to point out the unsupported assertions; our readers will at once perceive them.

"When the blood globules once reach these varicose sacs, it is highly improbable that they should ever proceed again to the pulmonary system. The extreme tenuity to which the vessels arrive, and their rapid growth, shows how readily parts adapt themselves to circumstances. Instead of depurating the blood, the vessels, by a change of state and increase of their contents, separate pus, as vicarious of bile which is evacuated through the natural channel of the biliary secretion. Dr. Cruveilhier of Paris mentions that, in enlarged ducts of a dog, he found only blood; a case, probably, in which the rupture of varicose capillaries had given rise to hemorrhage. That undue aeration of the blood, whether from extensive pulmonary disease, as in the case of phthisis cited, or from its detention in varicose capillaries, or any other cause, will give rise to the purulent degeneration of the globules, I do not doubt. In persons in whose blood pus has formed during a residence in hospitals, an event of frequent occurrence, all traces of it have disappeared after two or three days spent in a purer atmosphere. On the same principle blood detained in varicose capillaries, instead of being purified by secretion, and afterwards transmitted to the lungs, would degenerate into pus. But how this new form of the pus globule is produced must at present be left undetermined, for no other instances of a secretion of pus from venous blood have been collected in aid of the inquiry." 11.

Dr. Hake adds:—

"There were no traces of the ovate corpuscules in the urinary bladder; the chyle also was free from all trace of their presence. Although the blood offered no indications of their existence in the circulation, it presented appearances worthy of notice. In the liver, spleen, and kidneys, the blood globules were confluent; that is to say, had lost their individuality to a greater or less degree, and had run into each other, forming masses: a state of the blood which I have witnessed under other circumstances, as in fever, hæmorrhoids, &c.; and even in nervous affections, as mental inquietude, palpitation, &c. when there has been no other deviation from health. In the lungs the blood globules were distinct, but often grouped into the form of the ovate corpuscules and their nuclei. In the liver the same groups appeared, but with the globules confluent. Here also the same large globules were observed as in the bile in the earlier stage of the disease.

There were distinctive differences among the ovate corpuscules found in the digestive organs. Those in the stomach appeared to have suffered partial digestion, and in the duodenum there was some variety in their form.

The capillary vessels of the stomach and duodenum which I had injected were aneurismal; and, in the parts which they supplied, there was observed a tendency to what in such cases is falsely denominated ulceration, a process which will be presently explained." 12.

*Hypothesis of Morbid Growths.*

Our author's conclusions are more sweeping than the bare facts observed by him would seem to warrant.

"The fact that all morbid productions, whether cancerous, tuberculous, osseous, or of other kinds, may not only co-exist, but be intimately mingled in structure; that these are found connected with purulent, serous, bloody, and other deposits;—this fact, considered by Béclard as one of the greatest sources of difficulty in the study of pathological anatomy, may, at the present era, be viewed as a collective proof that one and the same law presides over the development of all disease. Though the nature of this law has been unknown, authors have not failed to detect an intimate alliance between the various forms of carcinoma, so that scirrhus, cancer, medullary or gelatiniform sarcoma, have been called indifferently by the same name; while tubercle and melanosis have been united in the same class on the one hand, and melanosis and cancer on the other.

When Béclard, writing of cancer, said, 'This tissue has less consistence than scirrhus, though more than the cerebral substance; is of a milky white, interrupted, when cut, by red points formed by divided vessels: these, in fact, are very numerous; but their walls are very thin, and scarcely support the force of the injection,'—that author was not far off from the efficient cause of the disease; and, had his mind but been in search of that, the probability is that he would have detected the truth, which was thus within his reach.

In tubercle affecting the spleen, I have seen the capillary arteries aneurismal; that they are so in every form of tubercular growth, as well as cancerous, is yet to be proved; meantime, it appears more than likely that, while one common law operates in the nutrition of natural tissues by means of healthy vessels, the deviation from that law is attributable to diseased vessels.

That hemorrhage arises from the rupture of varicose capillaries, is seen in cancerous affections; that it springs from the same causes in all tissues, whether in the pulmonary, cerebral, or any other systems I would not express a doubt, but that research can immediately decide the proposition. These dilated and attenuated vessels, when the disease which they constitute is superficial, having no mechanical support, become gangrenous; a state which gives rise to the destruction of parts involved in malignant disease, and their consequent removal, not by absorption, but by the disintegration of the outer surface.

The capillaries in a healthy state are of a uniform diameter, their size being about that of the blood globule. From this fact it may be implied that, in all tissues which secrete pus, the capillaries are in a state of dilatation; for the pus globule is generally from one-third greater than, to twice the size of, the blood globule; consequently the capillaries which secrete the former must be of a proportionate diameter. This simple truth opens to investigation the state of the capillaries through the large class of scrofulous affections, venereal and other abscesses, and suppurating surfaces of every description." 13.

Such is the substance of Dr. Hake's paper. It would be uncandid to deny that the fact of varicose capillaries playing an essential part in morbid growths is an interesting, nay, probably, an important one. But it was always supposed that the capillaries must do the work, and their being enlarged or varicose is, to our apprehension, a natural à priori supposition too. How our ideas of the heterologous formations are to be simplified or enlarged, how we are to explain, or how to treat them, is enveloped (at least we dare to fear so) in the same obscurity as ever. That the mere dilatation of the capillaries in an organ plays a subordinate part in the phenomenon is rendered probable, to say the least of it, by the fact that, if the organ (capillaries and all) is extirpated, the disease too frequently returns elsewhere. Will not chemistry do more than the microscope to help us in this difficulty?

Dr. Hake ventures a few recommendations and hopes in conclusion, recommendations of a hesitating and hopes of a confident character.

"Should this minute inquiry bring other investigators into the same field of research, it is recommended that the examination of the capillaries should be omitted in no instance whatever, whether in aneurism of the large vessels, varix of the veins, hypertrophy and atrophy of organs, or even in diseases affecting the whole system, as inflammatory and febrile affections.

The scientific treatment of malignant diseases has yet to be discovered. It must be founded on experiments made on animals with medicines for the purpose of ascertaining the effect they produce on the capillaries, and on observations of the effects of remedies on the blood, a fluid evidently involved to a greater or less extent in the production of heterogeneous growths. In the mean time, in cases where excision of the disease is impracticable, but where the artery which is distributed to it, and the capillaries of which are aneurismal, is within reach, a ligature might be tried, without danger and with some hope of benefit. And this might be succeeded by venesection and transfusion, cautiously but regularly performed, an experiment which, in prudent hands, could be attended by no danger, but might lead to results which would advance our knowledge of practice in this most fatal class of diseases." 14.

